# Evidence of discrete yellowfin tuna (*Thunnus albacares*) populations demands rethink of management for this globally important resource

**DOI:** 10.1038/srep16916

**Published:** 2015-11-23

**Authors:** P. M. Grewe, P. Feutry, P. L. Hill, R. M. Gunasekera, K. M. Schaefer, D. G. Itano, D. W. Fuller, S. D. Foster, C. R. Davies

**Affiliations:** 1 CSIRO Oceans and Atmosphere Flagship, Castray Esplanade, Hobart 7000, Tasmania, Australia; 2 Charles Darwin University, Research Institute for the Environment and Livelihoods, Ellengowan Drive, Darwin 0909, Northern Territory, Australia; 3 Inter-American Tropical Tuna Commission, La Jolla, California, USA; 4 689 Kaumakani Street, Honolulu Hawaii. 96825, USA

## Abstract

Tropical tuna fisheries are central to food security and economic development of many regions of the world. Contemporary population assessment and management generally assume these fisheries exploit a single mixed spawning population, within ocean basins. To date population genetics has lacked the required power to conclusively test this assumption. Here we demonstrate heterogeneous population structure among yellowfin tuna sampled at three locations across the Pacific Ocean (western, central, and eastern) via analysis of double digest restriction-site associated DNA using Next Generation Sequencing technology. The differences among locations are such that individuals sampled from one of the three regions examined can be assigned with close to 100% accuracy demonstrating the power of this approach for providing practical markers for fishery independent verification of catch provenance in a way not achieved by previous techniques. Given these results, an extended pan-tropical survey of yellowfin tuna using this approach will not only help combat the largest threat to sustainable fisheries (i.e. illegal, unreported, and unregulated fishing) but will also provide a basis to transform current monitoring, assessment, and management approaches for this globally significant species.

International tuna fisheries, managed under the auspices of Regional Fisheries Management Organisations (RFMO), are central to global food security and underpin multi-billion dollar economic activity in the developed and developing world. International law and governance arrangements for these fisheries (i.e. United Nations Convention on the Law of the Sea and UN Fish Stocks Agreement), which straddle international boundaries and large proportions of ocean basins, assume that the areas of competence of each RFMO equate to single panmictic stocks of the major target species^1^. These current arrangements largely reflect geo-politics, the post-WWII development pattern of industrial tuna fisheries and a reliance on fisheries dependent sampling to examine population structure and connectivity within and between RFMOs. Hence, the validity of this “unit stock” assumption and the appropriateness of current assessment and management arrangements are largely untested.

Until recently, whether population genetic approaches (e.g. allozymes, mtDNA, and DNA microsatellites) had sufficient discriminating power to adequately test this assumption has been constantly debated^2^,^3^,^4^,^5^. Developments in next generation sequencing approaches have overcome limitations of previous technology and delivered substantial increases in the power of genetic methods to detect population differentiation in marine species^6^,^7^,^8^,^9^,^10^,^11^. In particular, new high-throughput sequencing has low development costs and shows great promise for identifying co-dominant single nucleotide polymorphism (SNP) markers that can be used to distinguish among populations at scales relevant to fisheries assessment and management^8^,^12^. These methods have the potential to test the “single stock” paradigm for highly migratory stocks and provide the technical foundation for global chain of custody and provenance systems necessary to improve accuracy of catch reporting and curb Illegal, Unregulated, and Unreported (IUU) fishing. Here we address these issues with a focus on yellowfin tuna (*Thunnus albacares*) in the Pacific, where it is the second-most important species in the world’s largest tuna fishery^2^,^13^.

Yellowfin tuna is distributed in a continuous pan-tropical band 45 degrees north and south of the equator, from the Gulf of Mexico in the western Atlantic eastward to the coast of the Americas in the Pacific Ocean. It is now recognized as a single species, although it was originally classified into seven sub-species based primarily on morphological variation^14^. Despite its demonstrated capacity for individual large-scale movements, tagging studies of yellowfin tuna in the Pacific Ocean suggest dispersal on the order of hundreds, rather than thousands of kilometers^15^,^16^,^17^. Morphometric and meristic differences have also been demonstrated for yellowfin tuna among regions of the Pacific: across both latitudinal and longitudinal gradients^18^,^19^. Groups of yellowfin tuna examined from isolated regions across the Pacific are morphometrically distinguishable and phenetic relationships reflect geographic origin^19^. These differences have been attributed to restricted movements, limited mixing, and environmental variation^19^.

Genetic studies of yellowfin tuna stock structure in the Pacific using mitochondrial DNA (mtDNA), allozyme, and nuclear DNA (nDNA) microsatellite markers have been inconclusive^5^,^20^,^21^. Results were generally consistent with yellowfin tuna representing a single panmictic population, with the observed local morphological discrimination driven by environmental heterogeneity that either influences phenotypic plasticity via gene expression, or is locally selected following spawning. The pattern is also consistent, however, with selection at a limited number of loci that are not easily identified using low throughput and limited resolution profiling technology. However, allele frequencies at one of the allozyme loci examined, GPI-A (PGI-F) in two independent studies, demonstrated a sharp East-West cline across the Pacific^5^,^21^. This result suggested some population heterogeneity and appeared to be temporally stable across decades^5^. Nevertheless, current assessment and management arrangements in the Pacific effectively treat yellowfin tuna as two single stocks associated with the two Pacific tuna RFMOs (i.e. Inter-American Tropical Tuna Commission; and Western and Central Pacific Fisheries Commission).

The current study investigated the population structure of yellowfin tuna among three distantly separated locations across the Pacific Ocean (Fig. 1). We demonstrate that there are at least three genetically discrete populations and that individuals can be assigned to their source population with a high degree of confidence. These results demonstrate that the panmictic assumption underpinning current management by tuna RFMOs is incorrect and requires re-assessment. An urgent next step is a global survey of tuna stocks in the major ocean basins to re-examine previous biological assumptions and, as necessary, inform development of more appropriate spatial management arrangements.

## Results

### SNP genotyping

The DArTsoft14 pipeline delivered a total of 14,412 SNPs (Supplementary Table 1). This number was reduced to 6217 loci (call rate >90% and MAF >5%). No significant departures from Hardy Weinberg equilibrium were found in any of the 6217 loci tested within each sampling location. Among the 6217 SNPs that passed all quality control filters, a total of 215 SNPs were identified as outlier loci putatively under positive selection (hereafter referred to as the LUPS-dataset) and a total of 5054 SNPs were identified as putatively neutral (hereafter referred to as the NL-dataset).

### Population structure and assignment

Global measures of genetic differentiation G, as computed with Genepop, were highly significant for both the NL and LUPS datasets. Pairwise G statistics for all population pairs were highly significant for the LUPS-dataset, but not for the NL-dataset. The contrast between global and pairwise measures of genetic differentiation suggests the existence of a relatively weak genetic structure as inferred from neutral markers.

Neither the PCA nor the STRUCTURE analysis of the NL-dataset revealed any pattern or presence of genetic heterogeneity (Supplementary Fig. 1). In contrast, both the PCA and the STRUCTURE analysis carried out on the LUPS-dataset clearly distinguished each location as a distinct cluster (Supplementary Fig. 2 and Fig. 2) the first round of STRUCTURE analysis of the LUPS-dataset supported the existence of two different clusters, one including the fish from Baja California and one including the fish from Coral Sea and Tokelau (Fig. 2A). All membership q values were above 0.8 except for one individual from Coral Sea (q = 0.74) and four individuals from Baja California (0.76 < q < 0.79). A second level of structure was found within samples from Coral Sea and Tokelau (Fig. 2B). Indeed, the second round of analysis distinguished two clusters with all the fish assigned to their original sampling location with q values above 0.8. No further clustering was found within the Baja Mexico samples in the second round of analysis or within the Coral Sea and the Tokelau samples in the third round of analysis. Therefore, the finest population structure revealed by the ‘hierarchical STRUCTURE analysis’ consisted of three populations, each one matching with a sampling location (Fig. 2).

An analysis to determine the minimal SNP panel required for discriminating among the three populations estimated a panel of ~18 SNPs would provide a probability of 99.9% of correctly assigning provenance of an individual to the correct population (Fig. 3). This level of correct assignment far exceeds that when random genotypes are considered, which require ~55 (±9.8) SNPs per panel to reach the same probability of assignment (Fig. 4).

## Discussion

Here, we provide the first unequivocal evidence of genetically distinct populations of yellowfin tuna at ocean basin-scale using SNP markers. Importantly, we have demonstrated that, with a small sub-set of these markers, provenance of individuals can be assigned with a high degree of confidence. This provides the foundation for practical, cost-effective approaches for analysis of population structure, provenance, and chain-of-custody systems for these multi-billion dollar common resources.

The implications of these findings for assessment and management of global tropical tuna populations are substantial and wide ranging. Contemporary assessment and management is based on the assumption that the management unit being assessed is a single reproductive population with mixing of spawning populations (e.g. stock assessments in the Pacific Ocean assume eastern and western stocks of skipjack (*Katsuwonus pelamis*), yellowfin (*T. albacares*), and bigeye (*T. obesus*) tunas^22^,^23^. This east-west division largely reflects historical development of the fisheries, evidence of large-scale individual movements from tagging (implying potential for full mixing), and the fact that results from earlier genetics studies failed to provide evidence of population structure within these large areas (e.g.^24^). These same assessments do incorporate spatial structure to accommodate differences in fisheries characteristics (e.g. catch, selectivity, gear), fish biomass, and connectivity among regions within the assessment area (i.e. estimated adult movement and recruitment)^1^,^25^; yet the fundamental population dynamics that determine productivity of the stock and, hence, sustainable yields, are assumed to be homogeneous across the stock. The results presented here require that these fundamental assumptions be reconsidered and raise the real potential for multiple, independent assessment and management units within the current RFMO jurisdictions.

The discrete nature of the populations and the high probability of assignment, demonstrate that the yellowfin tuna from the three locations sampled in this study are likely to be reproductively isolated units. This implies that their response to fishing will be independent, and potentially different, depending on their relative productivities and levels of fishing mortality experienced. In particular, the evidence of discrete reproductive populations between the Coral Sea and Tokelau, strongly suggests that yellowfin tuna in these two regions within the WCPFC convention area should be assessed and, potentially, managed independently. These two areas reflect the nature of the contrast in the fisheries within the WCPFC jurisdiction: Tokelau, located in the high catch (50–150,000t/yr), mixed gear (purse-seine and longline), equatorial band, and the Coral Sea, low catch (5–10,000t/yr), higher latitude, longline only fishery. To assess these fisheries independently would require a more comprehensive, systematic genetic survey across the range of yellowfin tuna within the Pacific to determine number of populations and define the appropriate units for assessment and, if appropriate, management. Depending on the degree of mixing on feeding/fishing grounds, such a multi-population system may require catch-sampling programs to provide estimates of the proportion of different populations in catches from each area. Given the high probability of assignment of provenance using the markers under selection, this (assigning probabilistic sources of catches) is now feasible, and cost-effective, and could be implemented through at-sea or port sub-sampling of catches.

Results from tagging data and genetic markers from previous studies provided evidence indicative of east-west population structure of yellowfin tuna across the Pacific Ocean, which was temporally stable over several decades^5^,^19^,^21^,^26^. Other genetic stock structure investigations of yellowfin tuna have provided results ranging from inconclusive, i.e. no structure^24^,^27^,^28^, to presence of some heterogeneity^20^,^26^,^29^,^30^. In the cases where heterogeneity has been demonstrated (e.g.^20^,^26^) the markers lacked sufficient resolution to unambiguously assign provenance at the individual level. Similarly, the analysis of neutral markers in this study did not provide sufficient resolving power to demonstrate population heterogeneity. This is consistent with current genetics theory for large populations of highly mobile marine species with potential for high levels of gene flow and low rates of genetic drift^31^,^32^,^33^,^34^. The pattern of our results, i.e. eastern-central-western population heterogeneity for markers putatively under positive selection, however, are consistent with the temporally stable, population structure postulated in the study of Ward *et al.*^5^ and demonstrates these are highly likely to be reproductively isolated populations. This suggests that the distinct, multiple-population structure demonstrated here is likely to be present across the global distribution of yellowfin tuna^18^,^19^, with similar implications for the assessment and management of other large pelagic species.

The definitive nature of the population structure revealed here and capacity to define provenance with a small number of markers (<20) clearly demonstrates the power and efficiency of this new approach, even with the modest number of fish available to this study. Determining the specific implications for assessment and management of yellowfin tuna in the Pacific, and other oceans, requires a systematic global survey to provide a comprehensive description of population structure and associated estimates of connectivity. The (relatively) low cost and rapid rate of processing of this approach mean that such a survey could be completed within a matter of years, given sufficient international collaboration. Furthermore, the very high probability of assignment, combined with low unit cost, high through-put, inter-laboratory repeatability, and speed of processing associated with the genotyping-by-sequencing approaches suggest it is also well suited to large-scale, forensic screening required for chain of custody schemes and detecting and deterring activities associated with IUU fishing, such as catch substitutions. These results clearly demonstrate the need to define and incorporate this finer scale population structure of yellowfin tuna, and other “highly migratory species” in both their assessment and management and, potentially, allocation of access rights for these globally important resources.

## Methods

### Sample collection

**Figure 1:**
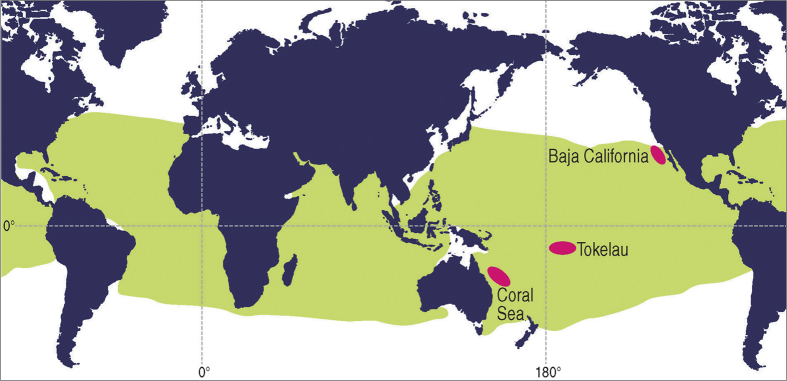
Approximate sampling locations (pink ovals) of fish used in the present study (Coral Sea, Tokelau, Baja California) overlaid on global distribution of yellowfin tuna (green shading). Source: Food and Agriculture Organization of the United Nations, FAO Species Catalogue. Vol. 2. Scombrids of the world. An annotated and illustrated catalogue of Tunas, Mackerels, Bonitos and related species known to date. Collette, B.B. & C.E. Nauen 1983. FAO Fisheries Synopsis, (125)Vol.2:137, [http://www.fao.org/fishery/species/2497/en]. Reproduced with permission. (Figure produced in Photoshop using Mountain High Maps® Copyright © 1993 Digital Wisdom®, Inc., 6.04 World (Gall projection) as the base map).

**Figure 2:**
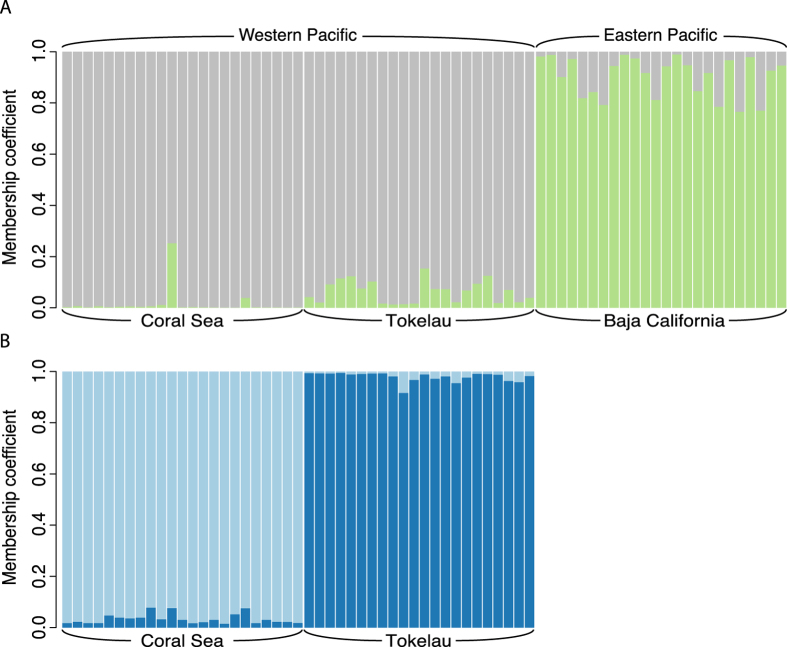
Results from analysis using STRUCTURE, indicating probability of membership coefficient of individuals to each of the sampling locations based on the analysis of loci putatively under positive selection. Panel-(**A**) 1st round of STRUCTURE analysis. Panel-(**B**) 2nd round of STRUCTURE analysis.

**Figure 3:**
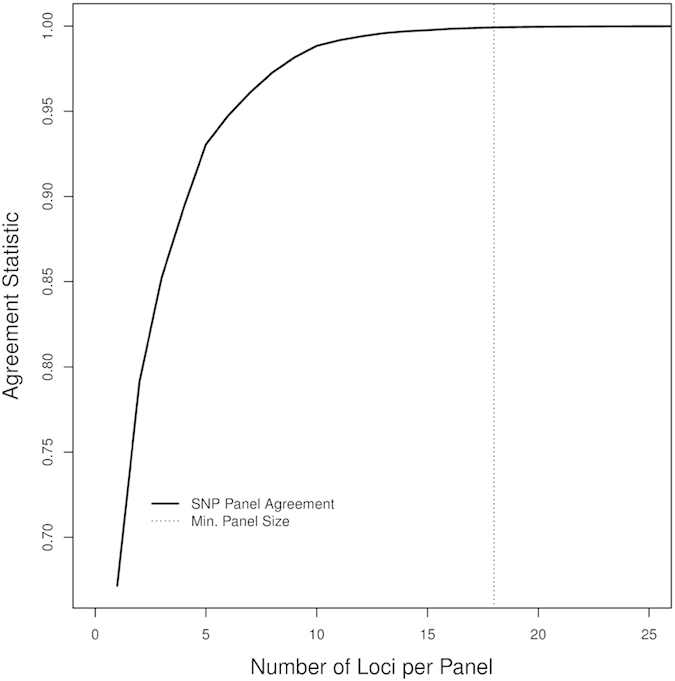
SNP panel efficacy demonstrated by plotting the agreement statistic (vertical axis) as a function of SNP loci included per optimal panel (horizontal axis). The vertical line gives the minimum panel size to reach an assignment agreement of 0.999.

**Figure 4:**
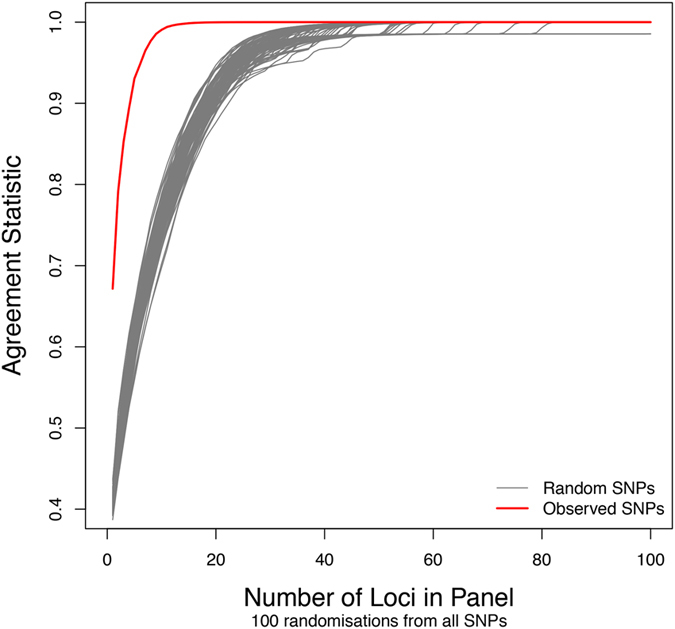
SNP panel efficacy based on the 100 randomly generated genotype data sets (grey plot lines) and demonstrated by plotting the agreement statistic (vertical axis) as a function of SNP loci included per optimal panel (horizontal axis). The red line is the agreement statistic for the real data.

Individual yellowfin tuna tissue biopsies were obtained from dead animals from commercial catches on board vessels fishing in areas located in the western (n = 23, Coral Sea, 22 °S_155 °E, February 2013), central (n = 22, Tokelau, 9 °S_172 °W, June 2012), and eastern (n = 24, Baja California, 31 °N_117 °W, December 2010) Pacific Ocean (Fig. 1). Fish ranged from 50 to 150 cm (fork length) covering multiple year classes per sampling region. Samples of white muscle were obtained close to the main dorsal fin and preserved in RNAlater® (Life Technologies) for shipment to laboratory for DNA extraction. Total genomic DNA was isolated using a modified CTAB procedure^35^.

### DArTseq genotyping

DArTseq™ represents a combination of DArT complexity reduction methods and next generation sequencing platforms^12^,^36^,^37^,^38^. This represents a new implementation of sequencing complexity reduced representations^39^ and more recent applications of this concept on the next generation sequencing platforms^40^,^41^. Similar to DArT methods based on array hybridisations, the technology is optimized for each organism and application by selecting the most appropriate complexity reduction method (both the size of the representation and the fraction of a genome selected for assays). Four methods of complexity reduction were tested in tuna (data not presented) and the PstI-SphI method selected. DNA samples were processed in digestion/ligation reactions principally as per^12^ but replacing a single PstI-compatible adaptor with two different adaptors corresponding to two different Restriction Enzyme (RE) overhangs. The PstI-compatible adapter was designed to include Illumina flow cell attachment sequence, sequencing primer sequence and “staggered”, varying length barcode region, similar to the sequence reported by Elshire *et al.*^41^. The reverse adapter contained a flow cell attachment region and an *Sph*I-compatible overhang sequence.

Only “mixed fragments” (PstI-SphI) were effectively amplified by PCR. PCR conditions consisted of an initial denaturation at 94 °C for 1 min followed by 30 cycles of 94 °C for 20 sec, 58 °C for 30 sec and 72 °C for 45 sec, with a final extension step at 72 °C for 7 min.

After PCR equimolar amounts of amplification products from each sample of the 96-well microtiter plate were bulked and applied to cBot (Illumina) bridge PCR followed by sequencing on an Illumina Hiseq2000. The sequencing (single read) was run for 77 cycles.

Sequences generated from each lane were processed using proprietary DArTseq analytical pipelines. In the primary pipeline, the FASTQ files were first processed to filter away poor quality sequences, applying more stringent selection criteria to the barcode region compared to the rest of the sequence. In that way the assignments of the sequences to specific samples carried in the “barcode split” step were very reliable. Approximately 2,000,000 sequences per barcode/sample were identified and used in marker calling. Finally, identical sequences were collapsed into “fastqcoll files”. The fastqcoll files were “groomed” using DArT PLD’s proprietary algorithm that corrects low quality base from singleton tag into a correct base using collapsed tags with multiple members as a template. The “groome” fastqcoll files were used in the secondary pipeline for DArT PLD’s proprietary SNP and SilicoDArT (presence/absence of restriction fragments in representation) calling algorithms (DArTsoft14). For SNP calling all tags from all libraries included in theDArTsoft14 analysis are clustered using DArT PL’s C++ algorithm at the threshold distance of 3, followed by parsing of the clusters into separate SNP loci using a range of technical parameters, especially the balance of read counts for the allelic pairs. Additional selection criteria were added to the algorithm based on analysis of approximately 1,000 controlled cross populations. Testing for Mendelian distribution of alleles in these populations facilitated selection of technical parameters discriminating well true allelic variants from paralogous sequences, In addition multiple samples were processed from DNA to allelic calls as technical replicates and scoring consistency was used as the main selection criteria for high quality/low error rate markers. Calling quality was assured by high average read depth per locus (Average across all markers at 59.1reads/locus, SD at 45.1 and Min at 3.3 reads/locus). As tuna reference genome is not yet available DArT has not performed alignment of tags/markers identified, hence mapping efficiency of reads against such reference could not be evaluated. For the current study only co-dominant SNP-DArT markers were used for population analysis.

### Data analysis

The data set used for population analysis from the DArTsoft14 pipeline consisted of 75 bp fragments containing one or more SNPs. When multiple polymorphisms were found on the same 75 bp fragment (RAD contig), a single SNP was randomly chosen to represent that locus to avoid linkage disequilibrium between close loci. Loci were further eliminated from population analysis by excluding loci with call rate (proportion of individuals scored for a locus) was lower than 90% and/or minor allele frequencies (MAF) lower than 5%. Departure from Hardy-Weinberg equilibrium (HWE) was tested for each locus within each sampling location using the “HWE.test.genind” function in the Adegenet R package^42^ and the false discovery rate method was applied to control for multiple comparison testing^43^.

Measures of genetic diversity, including observed and expected heterozygosities and allelic richness, were calculated for all SNPs that passed the quality filtering steps using the diveRsity R package (Table 1)^53^.

To identify putatively neutral loci and loci potentially located in genomic regions subject to positive selection, we used the F_st_ outlier approach developed by Beaumont & Nichols^44^ as implemented in LOSITAN^45^. A first LOSITAN analysis was done to remove potential selected loci previous to computing the initial mean F_st_. This F_st_ value was then used in a second LOSITAN analysis including all SNPs. The ‘Force mean F_st_′ option was selected in order to approximate a desired mean F_st_, by running a bisection algorithm over repeated simulations^45^. Each analysis ran for 1,000,000 simulations. We used a relaxed threshold P-value of 0.95 and a false error rate of 0.1 to select loci under positive selection (LUPS-dataset). Loci with P-values comprised between 0.05 and 0.95 were classified as neutral markers (NL-dataset). Other loci were removed from subsequent analyses.

Global and pairwise exact G tests of genetic differentiation were calculated using GENEPOP (v. 4.2 with default parameters)^54^ for both the LUPS and NL datasets.The presence of genetic structure was further tested using a principal component analysis (PCA) as implemented in the Adegenet R package and a Bayesian clustering method^49^ implemented in STRUCTURE (v. 2.3.4). We conducted a ‘hierarchical STRUCTURE analysis’ following the procedure described by Vähä *et al.* 46. For each round of analysis, the true number of clusters (K) was determined using the ad hoc statistic ΔK^47^ calculated using the online version of STRUCTURE HARVESTER (v. 0.6.93)^48^. As the ΔK method cannot find the best K when the true K = 1, we also examined individual assignment patterns and the hierarchical STRUCTURE analysis was stopped when no individuals would be assigned to any given cluster with a q value >0.8 in the subsequent round. Ten independent runs were executed for each K = 1–5. The admixture model with correlated allele frequencies among populations^49^ was chosen to analyze the LUPS-dataset. The NL-dataset was analyzed with the same model but setting sampling locations as priors, which can help with inferring clusters when population structure is weak^50^. For each analysis the MCMC was run for 500,000 repetitions, including a burn-in period of 200,000 repetitions. The burn-in period was defined so that each parameter had reached stationarity according to preliminary analyses.

The minimum number of SNPs required to distinguish the three populations was determined for the LUPS-dataset using forward selection (see^51^ for a review of selection methods and^52^ for a genetic application). This process adds the single SNP from the LUPS-dataset that best complements the previously identified set of SNPs. Panel selection starts with an empty set. The ability of a SNP to discriminate the populations is based on the conditional probability of the fish’s population membership, given that it must belong to one of the identified populations. The conditional probability can be considered as a soft, or probabilistic, classification. The final statistic combines the predicted membership and the actual membership and is defined as the sum (over fish and populations) of the product of the population membership and the conditional probability of membership. This agreement statistic approaches one if the conditional probabilities match the real population structure, and is zero when there is no agreement. Recalculating the allele frequencies without including a specific fish’s SNP data breaks the dependence between that fish’s SNP data and the populations’ allele frequency profiles. The number of SNPs required is defined to be the number of SNPs needed to give an agreement statistic of 0.999, roughly so that 99.9% of fish are correctly delineated.

There are just over 6000 SNP markers used in this study. There is the possibility that the SNP markers we identified were actually a subset that randomly partitioned among the three populations. However, we note that, if sufficient loci are examined, population differentiation can potentially be detected at random. However, this requires panels of many more SNPs than were observed for the real data. We investigated this hypothesis by examining the number of random SNPs required for a discriminating subset of loci to effectively partition the three populations. We randomly generated 100 SNP data sets with the same allele frequencies as those in the observed data set. We then identified the set of SNPs that best delineated the three populations, as was done previously, and calculated the agreement statistic. This test demonstrated the loci selected for the STRUCTURE analysis were in fact putatively under selection and not due to a random effect of number of loci examined.

## Additional Information

**How to cite this article**: Grewe, P.M. *et al.* Evidence of discrete yellowfin tuna (*Thunnus albacares*) populations demands rethink of management for this globally important resource. *Sci. Rep.* **5**, 16916; doi: 10.1038/srep16916 (2015).

## Supplementary Material

Supplementary Information

Supplemental Dataset 1

## Figures and Tables

**Table 1:** Summary of genetic diversity indices inferred from 6217 loci. N, sample size; MNA, mean number of alleles; Ar, allelic richness; Ho, observed heterozygosity; He, expected heterozygosity.

Location	N	MNA	Ar	Ho	He
Baja California	24	1.977 (0.149)	1.925 (0.174)	0.232 (0.171)	0.234 (0.142)
Tokelau	22	1.975 (0.155)	1.936 (0.174)	0.293 (0.210)	0.259 (0.144)
Coral Sea	23	1.979 (0.144)	1.931 (0.167)	0.225 (0.158)	0.234 (0.140)

Standard deviation in parentheses.
