# Supplementary material for: Evidence of discrete yellowfin tuna (Thunnus albacares) populations demands rethink of management for this globally important resource
Source: Sci Rep. 2015 Nov 23;5:16916. doi: 10.1038/srep16916 (PMC4655351; doi:10.1038/srep16916)

Supplementary Information

Evidence of discrete yellowfin tuna (*Thunnus albacares*) populations demands rethink of management for this globally important resource.

P.M. Grewe, P. Feutry, P. L. Hill, R. M. Gunasekera, K.M. Schaefer, D. G. Itano, D.W. Fuller, S. D. Foster, and C.R. Davies.

Supplementary Figure 1. Principal component analysis of the neutral loci.

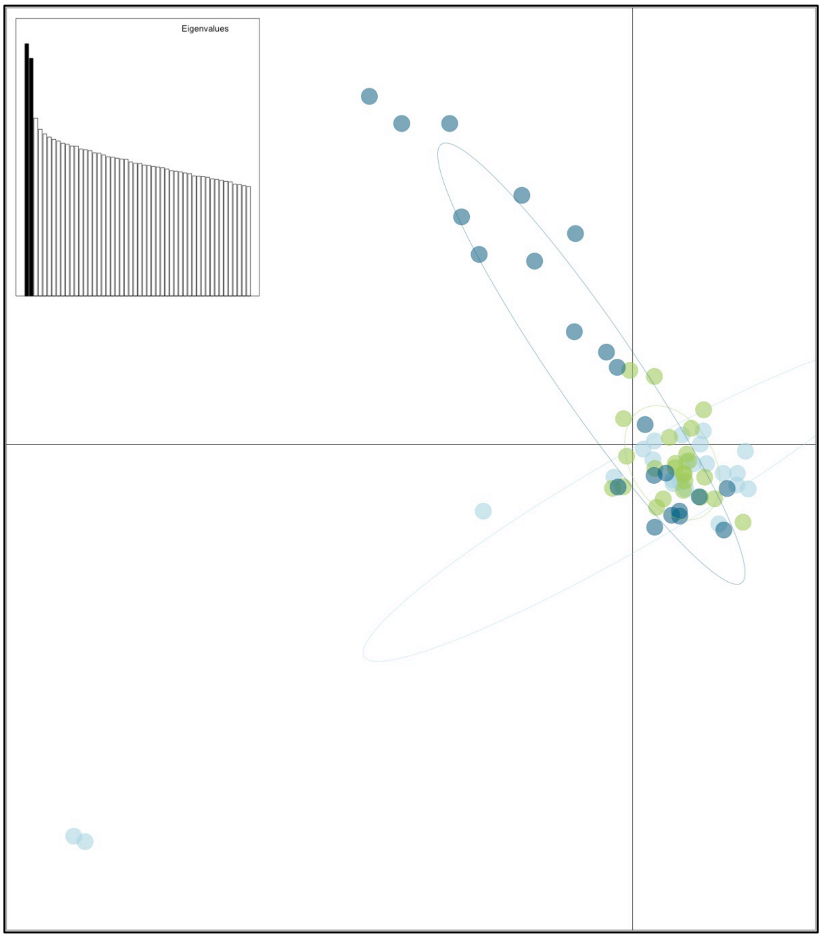

Supplementary Figure 2. Principal component analysis of the loci putatively under positive selection.

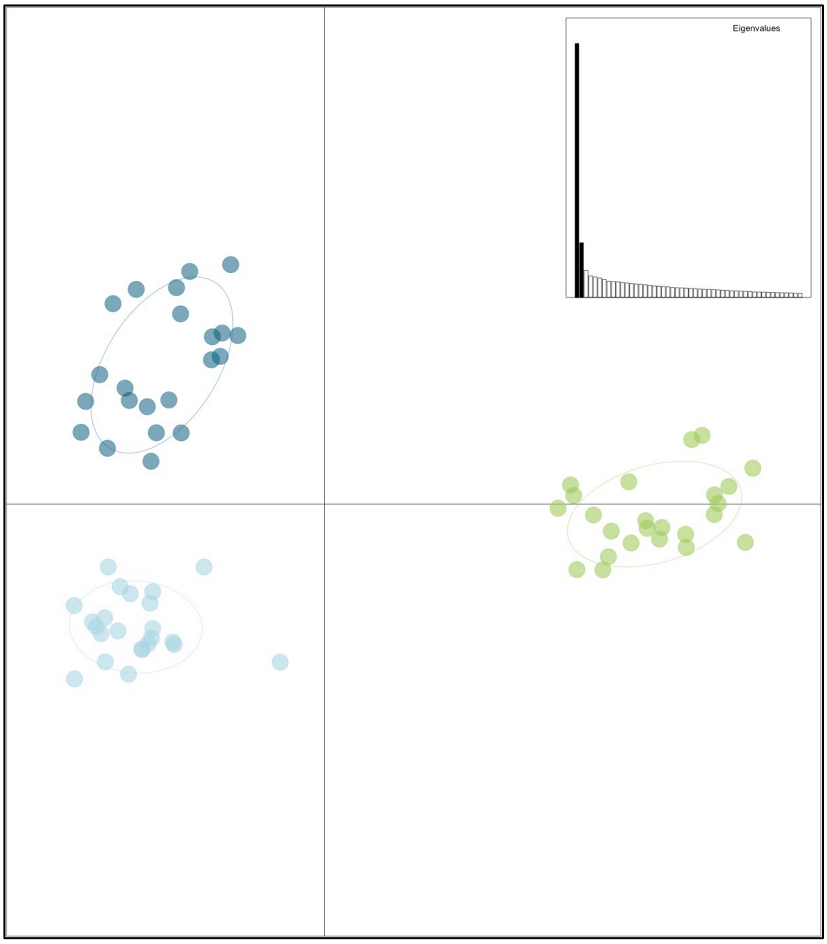

Supplement: Supplementary Information [file srep16916-s1.pdf]
